# Cell-specific tools for understanding behavior

**DOI:** 10.7554/eLife.106686

**Published:** 2025-04-14

**Authors:** Brandon L Holder, Stephane Dissel

**Affiliations:** 1 Division of Biological and Biomedical Systems, School of Science and Engineering, University of Missouri-Kansas City Kansas City United States

**Keywords:** central complex, neuropeptides, driver lines, cell types, sleep, neurotransmitters, *D. melanogaster*

## Abstract

Novel tools that allow neuron-specific investigations of the structure controlling sleep regulation in fruit flies reveal the extent of neuronal heterogeneity.

**Related research article** Wolff T, Eddison M, Chen N, Nern A, Sundaramurthi P, Sitaraman D, Rubin GM. 2025. Cell type-specific driver lines targeting the *Drosophila* central complex and their use to investigate neuropeptide expression and sleep regulation. *eLife* **14**:RP104764. doi: 10.7554/eLife.104764.

How and when animals sleep is a vital process that is tightly regulated. Even slight inaccuracies in this control can be fatal, particularly in dangerous environments (Lima et al., 2005). To ensure survival, animals have evolved multiple interconnected neuronal circuits to appropriately regulate sleep and wake behaviors. However, when conflicting signals arise, such as when humans consume both caffeine and sleeping pills at the same time, these regulatory mechanisms produce contradictory signals for behavioral output.

Animal models with simpler neural systems, such as the fruit fly *Drosophila melanogaster*, are invaluable in helping disentangle the various neuronal circuits controlling sleep across different species. In these insects, the central complex is a key brain region that regulates multiple behavioral processes including sleep (Pfeiffer and Homberg, 2014) and consists of approximately 2,800 neurons divided into 257 cell types (Hulse et al., 2021). This region is at the intersection of numerous sensory inputs and motor outputs.

Experimental tools exist to study neurons within the central complex, allowing researchers to examine the chemical messengers they release – such as neurotransmitters and neuropeptides – and how they affect neuron behavior. However, these methods often only allow large numbers of neurons to be manipulated, which may result in unreliable data, especially if neurons within the group are heterogeneous and have opposing functions (Figure 1A). Therefore, developing tools that allow single neurons or single types of neurons to be interrogated will allow researchers to convincingly assign behavioral outputs to specific neurons and cell types. Now, in eLife, Gerald Rubin and colleagues at Janelia Research Campus and California State University – including Tanya Wolff as first author – report generating tools that allow single types of neurons to be isolated and manipulated within the central complex of fruit flies (Wolff et al., 2025).

**Figure 1.**
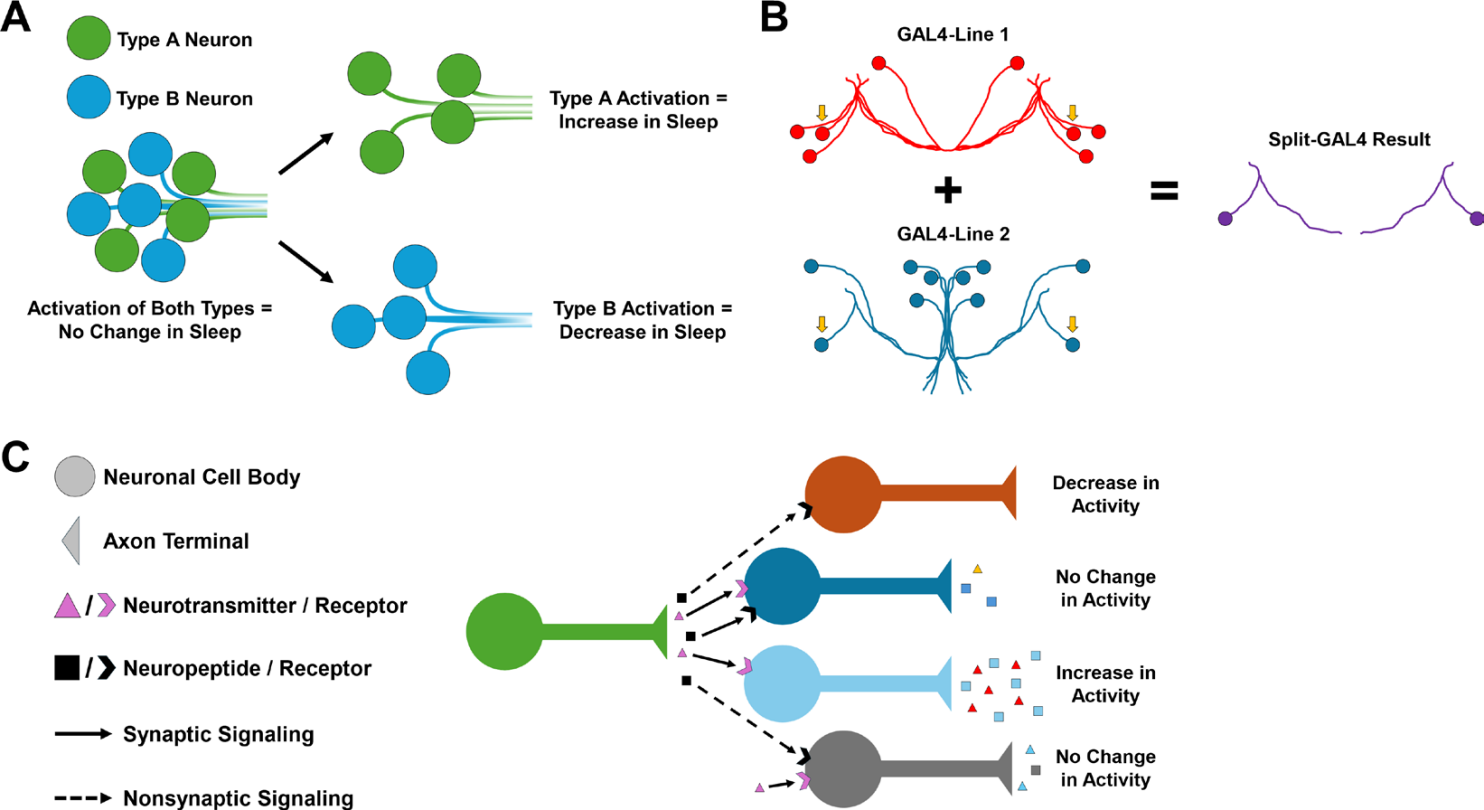
Neuronal heterogeneity impacts the behavioral output of neural circuits. (**A**) Schematic showing how the activation of a heterogeneous population of neurons can produce no change in a behavior, such as sleep, especially if neurons within the group have opposing functions. Activating specific cell types such as Type A (green) and Type B (blue) separately may lead to behavior changes, such as an increase or decrease in sleep. However, if both are activated at the same time, these opposing functions may cancel each other out and cause no change in sleep behavior. (**B**) Schematic of the Split-GAL4 system. This system relies on a yeast transcription factor known as GAL4, which when inserted into the fruit fly genome can be used to manipulate the behavior of neurons in specific cells by controlling gene expression. The split version of GAL4 is separated into two domains, which are both required for gene expression to be modified. This means that only cells that express both domains (indicated by the yellow arrows) will be modified, making it easier to pinpoint specific cell types. (**C**) Different combinations of neurotransmitter, neuropeptide, and neuropeptide receptor expression can fine-tune neuronal activity and generate different neuronal responses in postsynaptic cells, even within neurons of the same group. For example, non-synaptic signaling by a neuropeptide (black) may cause a decrease in neuronal activity on its own (orange). However, when combined with synaptic signaling by a neurotransmitter (pink), this may lead to no change in neuronal activity (gray).

For this, the team used an advanced genetic technique known as the Split-GAL4 system, which allows gene expression to be controlled in individual or small groups of neurons (Figure 1B; Brand and Perrimon, 1993; Luan et al., 2006). By generating Split-GAL4 cell lines for nearly one half of all cell types in the central complex, Wolff et al. could control the expression of specific genes in each of these cell types in flies. This allowed them to ‘switch on’ certain fluorescent labels in specific cells, which when combined with fluorescent in situ hybridization – a technique that allows researchers to visualize where genes of interest are expressed – revealed the gene expression patterns of several neuromodulators and receptors across different cell types.

The tested genes included signaling molecules which act on neurons both locally (i.e. neurotransmitters such as acetylcholine, glutamate, and dopamine), and over long distances (i.e. neuropeptides). This approach revealed immense variability in the neurotransmitters, neuropeptides and neuropeptide receptors expressed in the neurons of the central complex, even in neurons that were thought to be similar, such as those in the dorsal fan-shaped body. These findings underscore both the neurochemical heterogeneity and complexity of the central complex, while stressing the importance of studying neuropeptides just as intensely as classical neurotransmitters (Figure 1C).

Wolff et al. also used the fly lines to perform a behavioral analysis of sleep by pinpointing cells whose experimental activation had a strong impact on sleeping and activity patterns. This revealed neurons within the central complex that promote wake and others that promote sleep, including several not previously identified as regulating sleep. Interestingly, some neurons that appear to be morphologically related or project to the same structure had different effects on sleep when activated experimentally.

Finally, Wolff et al. examined the link between the central complex and the circadian clock, the neural system that generates approximately 24 hour rhythms in sleep and wakefulness in response to the light-dark cycle. Using a map of neural connections in the brain (Dorkenwald et al., 2024; Hulse et al., 2021; Schlegel et al., 2024), the team identified many wired pathways between the central complex and the circadian clock. However, Wolff et al. also rationalize that their experimental results support the notion that indirect neuropeptide pathways also exist between the two, based on extensive expression of both neuropeptides and receptors. Consequently, neuropeptides may modulate distant neurons where no physical connection is evident using the map of neural connections, which adds complexity to these already intricate systems.

Taken together, the findings reveal extensive heterogeneity in the neurons of the central complex and identify unique neurotransmitter, neuropeptide, and neuropeptide receptor expression that collectively fine-tune the response of individual neurons, even within the same named structure (Figure 1C). This is in line with previous work, for instance studies surrounding the dorsal fan-shaped body of the central complex and its disputed role in sleep regulation in *Drosophila* (De et al., 2023; Hasenhuetl et al., 2024; Jones et al., 2023; Jones et al., 2025; Keleş et al., 2025). The findings of Wolff et al. underscore the importance of using cell-specific tools to overcome neuronal heterogeneity in future behavioral studies and pave the way for future intricate analyses.
